# Effects of a 12-week supervised resistance training program, combined with home-based physical activity, on physical fitness and quality of life in female breast cancer survivors: the EFICAN randomized controlled trial

**DOI:** 10.1007/s11764-022-01192-1

**Published:** 2022-03-22

**Authors:** Alberto Soriano-Maldonado, David M. Díez-Fernández, Alba Esteban-Simón, Manuel A. Rodríguez-Pérez, Eva Artés-Rodríguez, Miguel A. Casimiro-Artés, Herminia Moreno-Martos, Antonio Toro-de-Federico, Nur Hachem-Salas, Cecilie Bartholdy, Marius Henriksen, Antonio J. Casimiro-Andújar

**Affiliations:** 1 Department of Education, Faculty of Education Sciences, University of Almería, Almería, Spain; 2 SPORT Research Group (CTS-1024), CERNEP Research Center, University of Almería, Almería, Spain; 3 Area of Statistics and Operative Research, Department of Mathematics, Faculty of Sciences, University of Almería, Almería, Spain; 4 Realtrack Systems S.L, Almería, Spain; 5 Servicio Andaluz de Salud, Unidad de Gestión Clínica Almería Periferia, Distrito Sanitario, Almería, Spain; 6 Servicio Andaluz de Salud, Unidad de Gestión Clínica Ciudad Jardín, Distrito Sanitario, Almería, Spain; 7 Servicio Andaluz de Salud, Unidad de Gestión Clínica Mediterráneo-Torrecárdenas, Distrito Sanitario, Almería, Spain; 8 The Parker Institute, Bispebjerg-Frederiksberg Hospital, University of Copenhagen, Frederiksberg, Denmark

**Keywords:** Resistance training, Breast cancer, Muscular strength, Cancer-related fatigue, Health-related quality of life

## Abstract

**Purpose:**

This study assessed the effects of 12-week supervised resistance training combined with home-based physical activity on physical fitness, cancer-related fatigue, depressive symptoms, health-related quality of life (HRQoL), and life satisfaction in female breast cancer survivors.

**Methods:**

A parallel-group, outcome assessor-blinded, randomized controlled trial included 60 female breast cancer survivors who had completed their core treatments within the previous 10 years. Through computer-generated simple randomization, participants were assigned to resistance training (RTG; two sessions/week for 12 weeks plus instructions to undertake ≥ 10,000 steps/d) or control (CG; ≥ 10,000 steps/d only). Outcomes were evaluated at baseline and week 12. Muscular strength was assessed with electromechanical dynamometry. A standardized full-body muscular strength score was the primary outcome. Secondary outcomes included cardiorespiratory fitness, shoulder mobility, cancer-related fatigue, depressive symptoms, HRQoL, and life satisfaction.

**Results:**

Thirty-two participants were assigned to RTG (29 achieved ≥ 75% attendance) and 28 to CG (all completed the trial). Intention-to-treat analyses revealed that the standardized full-body muscular strength score increased significantly in the RTG compared to the CG (0.718; 95% CI 0.361–1.074, *P* < 0.001, Cohen's *d* = 1.04). This increase was consistent for the standardized scores of upper-body (0.727; 95% CI 0.294–1.160, *P* = 0.001, *d* = 0.87) and lower-body (0.709; 95% CI 0.324–1.094, *P* = 0.001, *d* = 0.96) strength. There was no effect on cardiorespiratory fitness, shoulder flexion, cancer-related fatigue, depressive symptoms, HRQoL, or life satisfaction. The sensitivity analyses confirmed these results.

**Conclusion:**

and implication for cancer survivors.

In female breast cancer survivors who had completed their core treatments within the past 10 years, adding two weekly sessions of supervised resistance training to a prescription of home-based physical activity for 12 weeks produced a large increase in upper-, lower-, and full-body muscular strength, while other fitness components and patient-reported outcomes did not improve.

Trial registration number.

ISRCTN14601208.

**Supplementary Information:**

The online version contains supplementary material available at 10.1007/s11764-022-01192-1.

## Introduction

Breast cancer is the most commonly diagnosed cancer type (~ 2.1 million new cases every year) and the leading cause of death in women worldwide [1]. In 2020, there were 2.3 million women diagnosed with breast cancer and 685,000 deaths globally [2]. In Spain, the incidence of breast cancer increased from approximately 26,000 new diagnoses in 2017 to over 32,000 in 2019 [3]. Current advances in early diagnosis and treatment have led to a significant reduction of breast cancer mortality [4]. For instance, in a developed country such as Canada, the net survival five-year estimates for women diagnosed with breast cancer was 87% [5]. In Europe, breast cancer mortality was estimated to be reduced by over 10% in 2020, except for Spain that has the lowest mortality rate (11.6 cases/100,000 inhabitants) in Europe [6].

The abovementioned reduction in breast cancer mortality over time denotes that an increasing number of women are living long after their initial cancer diagnosis and treatment, which implies facing many short-, mid-, and long-term treatment side effects. Consequently, addressing the management of the breast cancer-related side effects is of major clinical and public health interest. Common side effects following breast cancer includes lymphedema [7], cardiac toxicity [8], fatigue [9], depression [10], bone health issues, and obesity [11]. These problems, together with a significant loss of upper-limb mobility [12], loss of general muscular strength and muscle mass [13], and compromised life satisfaction and health-related quality of life (HRQoL) [14], should be monitored in the follow-up of breast cancer [15]. In particular, muscular strength during treatment has been reported to be 25% lower in the lower extremities and 12–16% lower in the upper extremities compared to healthy individuals [16]. Similar trends seem to occur regarding cardiorespiratory fitness [17, 18] and upper-limb function and mobility [12]. These tendencies might worsen in the absence of physical activity in the years following treatment [19].

Current guidelines for the management of breast cancer survivors include counselling regarding physical activity [15]. Consequently, for ethical reasons, any exercise-based clinical trial enrolling breast cancer survivors should ensure that all trial participants meet the international physical activity guidelines (at least 150 min per week [20]). In addition, structured exercise seems to benefit several of the side effects [21–24]. As a structured exercise form, resistance training has shown to enhance muscular function, body composition, and to some extent fatigue [25]. It is important to highlight that the loss of muscle function and strength compromises the functional ability to produce force during activities of daily living and increases the risk of physical disability and death [26]. In particular, breast cancer survivors who engage in resistance training show a 33% lower mortality risk [27] and this type of exercise has shown to be safe for limb-related issues such as lymphedema [28–30].

However, the exercise interventions in oncological exercise-based clinical trials, including breast cancer trials [31], are poorly reported [32]. In a recent systematic review, Neil-Sztramko et al. [31] concluded that “no studies of exercise in women with breast cancer attended to all principles of exercise training or reported all components of the exercise prescription in the methods, or adherence to the prescription in the results,” which precludes transparency, replicability, and comparisons across clinical trials in this population. The systematic review of Fairman et al. [33] revealed that resistance training prescription across exercise oncology studies is very heterogeneous and largely underdeveloped. The failure of prior research to apply the exercise principles may largely explain the heterogeneity observed across study outcomes in various systematic reviews and meta-analyses [22, 34–38]. This opens a window of opportunity for upcoming trials to correctly implement the exercise principles and adhere to the reporting guidelines for exercise trials [39], thus contributing to the development of resistance training guidelines in breast cancer survivors [31] and potentially being effective in increasing physical function and quality of life.

The aim of this clinical trial was to evaluate the effects of a 12-week supervised resistance training program combined with a prescription of home-based physical activity, compared with home-based physical activity alone, on muscular strength, physical fitness, cancer-related fatigue, depressive symptoms, health-related quality of life, and life satisfaction in female breast cancer survivors.

## Material and methods

### Design and protocol registration

The Ejercicio FIsico para supervivientes de CANcer de mama (EFICAN; in English, *physical exercise for breast cancer survivors*) randomized controlled trial is a parallel-group, randomized controlled trial prospectively registered (ISRCTN14601208) on August 1, 2019, before the enrolment of participants begun (August 12, 2019). A comprehensive description of the rationale and methodology has been published [40].

### Setting and eligibility criteria

The participants were recruited through local cancer-related associations, advertisements in local newspapers, and radio and social media including social networks and through referral from clinical oncologists from the Torrecárdenas University Hospital (Almería, Spain). Eligible participants were voluntary women aged 18–65 with a breast cancer diagnosis, who had completed their core treatments (surgery, chemotherapy, and/or radiotherapy) within the prior 10 years at the time of recruitment. The exclusion criteria included metastatic breast cancer, being scheduled for breast reconstruction in the following 6 months, presenting with any pathology that might contraindicate exercise, or being highly physically active (structured exercise > 300 min/week). This study was approved by the Almería Provincial Research Ethics Committee, Almería, Spain (ref: Ejercicio-CáncerUAL[98/2019]) on 31/07/2019.

### Procedures

Interested women filled out an online form with basic sociodemographic and disease-related information, and potentially eligible participants were invited for an in-person screening, where medical doctors (MD) assessed the inclusion and exclusion criteria and obtained participants’ informed consent. The enrolled participants attended the exercise laboratory at the University of Almería to complete the baseline assessments. This study adheres to the CONSORT guidelines [41]. The funding sources had no role in the study. All databases including personal information were collected by the principal investigators (AS-M and AJC-A) who were responsible for protecting confidentiality.

### Sample size

The sample size was calculated for muscular strength. A between-group difference in the change from baseline in upper-limb muscular strength of at least 6.9 kg would be considered clinically relevant [25]. Assuming a common standard deviation of 7.5 kg, a 90% power, an alpha error of 5%, and a potential dropout of 20%, 60 participants were recruited.

### Randomization, treatment allocation, and blinding

Each participant was randomized (1:1 ratio) either to a resistance training group (RTG) or a waiting list control group (CG). Before the participant’s recruitment, a blinded investigator (AS-M) created a computer-generated simple randomization sequence. Individual allocations were held in sealed, opaque, and consecutively numbered envelopes. After baseline assessments, a blinded member of the staff opened the envelopes in front of each participant and assigned them to the intervention groups. The data analyst and the primary outcome assessors were blinded to the participant allocation.

### Intervention

All the participants were requested to continue their habitual lifestyle and their eventual medications during the trial. All the study participants were requested to perform home-based physical activity defined as undertaking ≥ 10,000 steps per day [42, 43]. Compliance with this requirement was monitored through an activity bracelet (Xiaomi MiBand3, Xiaomio Inc., Pekin, China). The intervention period ranged from September 23, 2019, to December 13, 2019.

#### Resistance training group (RTG)

To maximize transparency and replicability, this exercise protocol follows the Consensus on Exercise Reporting Template (CERT) [39], and the comprehensive description of the intervention with the CERT checklist is published elsewhere [40]. Participants in the RTG performed two resistance training sessions per week (with ≥ 24–48 h recovery between sessions) for 12 weeks (a total of 24 sessions of 60 min), divided into two phases: phase 1 (i.e., familiarization) included two individual (1:1 ratio) training sessions per week for two weeks, where the exercise professional determined individual needs and limitations and the participants learnt basic movement patterns, and phase 2 included two group-based (four to six participants) training sessions per week that included a preparatory part (warm-up) with aerobic activity, mobility and stability exercises, a main part comprising circuit-based resistance training, and a cool down part with stretching, for 10 weeks. The starting level of each participant was set considering their baseline muscular strength and the work undertaken individually during phase 1.

The supervised training sessions were led by exercise professionals with a degree in Physical Activity and Sport Sciences and with specific training in exercise for breast cancer and < 1 year of experience. The exercise program was carried out in a fitness room at the Almería town hall (400 m^2^).

Supplementary Table S1 summarizes the design (i.e., periodization) of the resistance training program. Each session comprised 3 parts. Part 1 was a preparatory part of ~ 15 min, divided into 5 min of low-intensity aerobic activity (50–65% of the heart rate reserve) either on a treadmill or an elliptical trainer, two chest mobility exercises and two central stability (i.e., CORE) exercises (~ 5 min), and two scapulohumeral joint stability and two dynamic stability exercises (~ 5 min) [Supplementary Table S2]. The exercise intensity for part 1 was set at 3 out of 10 quantified through the OMNI Perceived Exertion Scale for Resistance Exercise (OMNI-RES) [44]. Part 2 (the main part) comprised a circuit of 4 dynamic resistance exercises (i.e., bilateral deadlift, bilateral seated row, bilateral squat, and bilateral seated bench press). Resistance training intensity was equivalent to 40–70% of one repetition maximum (1 RM) and was individually estimated so that participants progressively work from a training load that could be lifted 24 times (24 RM; approximately 40% of 1 RM) to a training load that could be lifted 12 times (12 RM; approximately 70% of 1 RM) throughout the full range of motion. Progressions generally occurred weekly. Although 60–70% of 1 RM is recommended in healthy adults to improve muscular strength [45], previous research has shown that moderate intensities (40–60% of 1 RM) can improve muscular power, strength, muscular size, and functional tasks even in older people [46]. Exercise intensity was individually quantified through the character of effort (CE; which represents the number of repetitions actually performed relative to the maximum number of repetitions that the participant could theoretically perform with a given load), as previously reported [47, 48], and participants were asked to report their subjective level of effort (after each exercise) using the OMNI-RES [44]. The CE was set so that participants self-selected the absolute load that allowed them to approximately perform a maximum number of possible repetitions (Supplementary Table S1) but performed half of the possible repetitions to maximize strength gains [49, 50] and minimize risks. For greater strength gains, participants were required to perform the concentric phase of each exercise at their maximum voluntary velocity [46, 51]. The resting periods between sets of a given exercise ranged from 1.5 to 3 min [46]. Part 3 consisted of a collective cooldown, including dynamic/static stretching of major muscle groups (i.e., pectoralis major, dorsal width, quadriceps, and hamstrings), and a general group evaluation of the session. The main exercises performed along the exercise program are presented in Supplementary Table S2, and a video library with all the exercises is freely available online as supplementary videos.

All the participants were requested to report any difficulties, limitations, or needs so that the intervention could be adapted to individual characteristics (Supplementary Table S2). The motivational strategies to maximize adherence and how adherence was collected are published elsewhere [40]. There were no nonexercise components for this intervention.

#### Control group (CG)

Participants assigned to the CG were requested to undertake ≥ 10,000 steps per day as home-based physical activity [42, 43] but were not offered participation in the resistance training program. For ethical reasons and to maximize participation, the participants assigned to CG had the opportunity to participate in the resistance exercise program once the trial was completed.

### Outcome measures

All outcome measures were assessed at baseline and at week 12 (after completing the intervention period). The baseline assessments were carried out during 14 days prior to the beginning of the intervention, and the follow-up assessments were conducted within 10 days following the intervention period. The principal investigators (AS-M and AJC-A) were responsible for the dataset.

#### Primary outcome measure: muscular strength

The peak isometric muscular strength (measured in N) was assessed with an electromechanical dynamometer (Dynasystem® Research, Symotech, Granada, Spain) [52, 53]. This device has shown high reliability (CV < 3%; ICC > 0.90) and high concurrent validity (*r* = 1.00; systematic bias < 13.9 N; random error < 52.1 N) for assessing peak force during the isometric mid-thigh pull (IMTP) test [54]. Each test was performed once, where maximal effort was requested during a 6-s trial. If the execution was not correctly performed (at the discretion of the evaluator), a new attempt was conducted after a rest period of ~ 3 min. Verbal stimulation was provided during testing to motivate the participants to achieve a maximum effort.

**Fig. 1:**
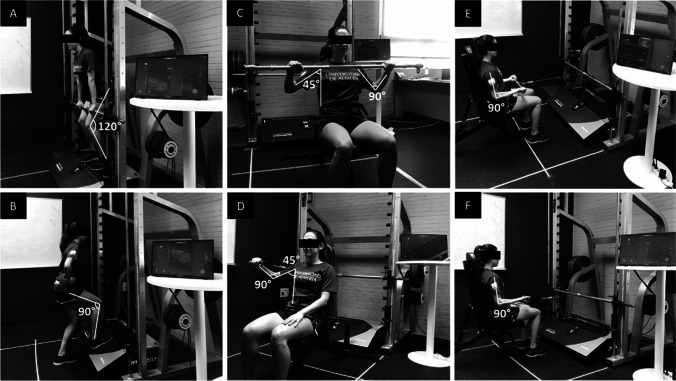
Graphical representation of the muscular strength assessment comprising the isometric mid-thigh pull test (**A**), the unilateral isometric knee extension in a closed kinetic chain at 90° (**B**), the bilateral isometric seated bench press (**C**), the unilateral isometric seated bench press (**D**), the bilateral isometric seated row (**E**), and the unilateral isometric seated row (**F**). Reprinted from Soriano-Maldonado et al. Medicine 2019;98:44(e17625), distributed under the terms of the Creative Commons CC-BY license (i.e. no permissions required)

The primary outcome measure was a standardized full-body muscular strength index, defined as the average of the normalized scores (*z*-score = [value-mean] / standard deviation) of the changes from baseline to week 12 in the two upper-body tests depicted in Fig. 1D and F, and the two lower-body tests depicted in Fig. 1A and B, and computed as indicated in the Supplementary file, page 4.

#### Secondary outcomes

##### Secondary muscular strength outcomes.

Changes from baseline to week 12 in upper-body muscular strength were assessed as the average of the standardized score (*z*-score = [value-mean] / standard deviation) of the change from baseline to week 12 in 2 different tests, including (i) the sum of the right and left unilateral isometric seated bench press (Fig. 1D) and (ii) the sum of right and left unilateral isometric seated row (Fig. 1F).

Changes from baseline to week 12 in lower-body muscular strength were assessed as the average of the standardized score (*z*-score = [value-mean] / standard deviation) of the change from baseline to week 12 in 2 different tests, including (i) the sum of the right and left unilateral isometric knee extension in closed kinetic chain at 90° (average of the right and left knees; Fig. 1B) and (ii) the IMTP test (Fig. 1A).

Changes from baseline to week 12 in the peak isometric strength for bilateral seated bench press (Fig. 1C) and bilateral seated row (Fig. 1E) were measured with the abovementioned electromechanical dynamometer; changes from baseline to week 12 in handgrip strength (of the affected and nonaffected arms) were assessed with a digital dynamometer (Model T.K.K.540®; Takei Scientific Instruments Co., Ltd., Niigata, Japan) where the best (out of two) trial performed with the affected and the best (out of two) trial performed with the nonaffected limb were averaged.

##### Cardiorespiratory fitness.

Cardiorespiratory fitness (i.e., maximum oxygen consumption [VO_2máx_; mL/min/kg]) was estimated through the Siconolfi step test as described elsewhere [40]. This test has been developed for use in epidemiological studies [55] and has been used in different populations [56, 57].

##### Shoulder flexion range of motion.

The range of shoulder flexion in a supine position was assessed (and measured in degrees) through digital goniometry (HALO Digital Goniometer, HALO Medical Devices HQ, Sydney, Australia) following the protocol described elsewhere [58]. Each participant performed two trials with each arm, and the average of each arm was used.

##### Cancer-related fatigue.

Cancer-related fatigue was assessed with the patient-reported Functional Assessment of Cancer Therapy-Fatigue (FACT-F) [59]. The scores range from 0 to 52, where a higher score indicates lower fatigue.

##### Depressive symptoms.

Depressive symptoms were assessed with the patient-reported Center for Epidemiologic Studies-Depression Scale (CES-D) [60]. The final score ranges from 0 to 60, where a higher score indicates greater depressive symptoms.

##### Health-related quality of life.

Health-related quality of life was assessed with the patient-reported Functional Assessment of Cancer Therapy-Breast (FACT-B) [61]. The global score ranges from 0 to 148 where higher scores indicate higher HRQoL.

##### Life satisfaction.

Life satisfaction was assessed with the Spanish version [62] of the patient-reported Satisfaction with Life Scale (SWLS) [63]. The final score ranges from 0 to 25, where a greater score indicates greater satisfaction with life.

### Data collection procedure

Both the baseline and the follow-up assessments were conducted at the Exercise Laboratory of the University of Almería. Each participant begun filling out a general sociodemographic questionnaire and all the patient-reported outcomes. Thereafter, anthropometric measures were taken in a private room, followed by the shoulder joint mobility, upper- and lower-body muscular strength, and cardiorespiratory fitness. This sequence was performed to minimize fatigue for the primary outcome assessment.

### Deviations from the original protocol

Due to recruitment difficulties, the eligibility criterion regarding time from completion of cancer treatments was changed from 5 to 10 years. In the protocol, several muscle strength variables were listed as primary outcomes. To avoid problems with interpretation of results from many primary outcomes, the primary outcome has been set as the standardized full-body muscular strength index alone; the muscular strength of the upper and lower body have been moved to secondary outcomes. The main analyses, initially set as per protocol, have been changed to intention to treat (ITT) to reduce the risk of bias.

### Statistical analysis

The distribution of the study variables was assessed through histogram and Q-Q plots. Descriptive characteristics are presented using the mean and standard deviation for quantitative variables and the number and frequency for categorical variables. The comparability of the groups at baseline was checked. The between-group differences in the primary and secondary outcomes were assessed through linear regression, including the baseline outcome value as a covariate. The effect sizes were assessed with Cohen’s *d* [64], and values of *d* equal to 0.2, 0.5, and 0.8 were considered small, medium, and large effects, respectively. We checked the homoscedasticity and linearity assumptions of the linear regression models, as well as the normality, non-multicollinearity, and non-autocorrelation of the residuals. The primary analyses were performed under the intention-to-treat principle using baseline observation carried forward (BOCF), and sensitivity analyses were conducted using per-protocol analyses (defined as ≥ 75% adherence to the intervention). A blinded investigator (AS-M) handled all hypothesis testing under the supervision of professional statisticians (EA-R and MAC-A). The main analyses were conducted with Stata v.16.1 (StataCorp LP., Texas, USA). Statistical significance was set at *P* < 0.05.

## Results

**Fig. 2:**
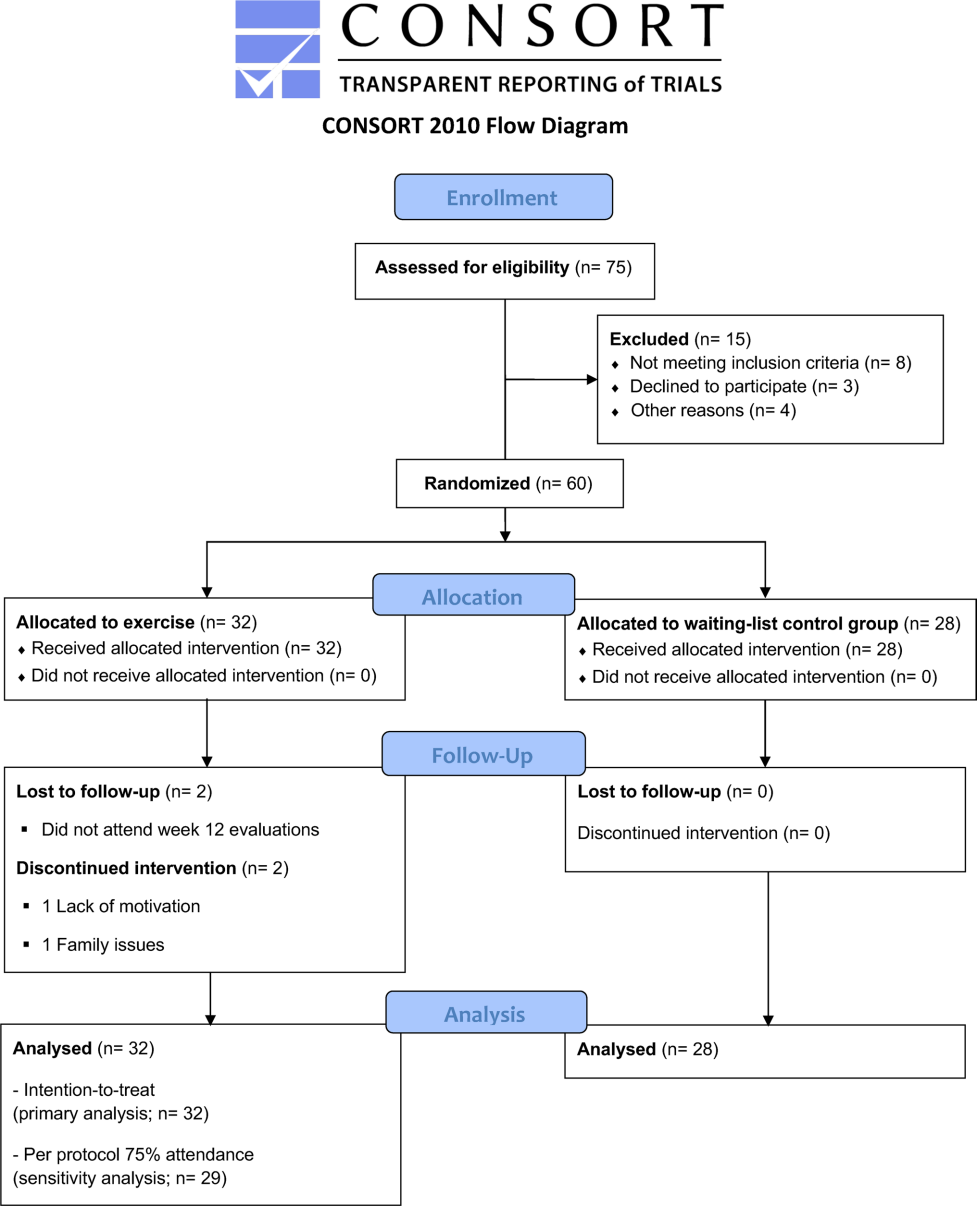
CONSORT flowchart of the study participants throughout the EFICAN randomized controlled trial

The CONSORT flowchart of the study participants throughout the study is presented in Fig. 2. A total of 75 potential candidates were screened, of which eight did not meet the inclusion criteria (one had metastasis, five finished core treatments > 10 years ago, one presented moderate chronic obstructive pulmonary disease, and one had a surgical procedure the week prior to baseline assessments), four declined to participate, and three reported other reasons such as lack of time. A total of 60 participants were randomized and allocated to the RTG (*n* = 32) and the CG (*n* = 28). Two participants in the RTG and none in the CG discontinued the intervention. The two participants in the RTG who discontinued the intervention were also lost to follow-up, whereas all the participants in the CG completed the trial. There were two adverse events in the RTG; one participant had a muscular overload in session 15 and one participant presented shoulder discomfort during session 17; both events persisted until the end of the intervention, although they did not impede the participants completion of the majority of the exercises (except those directly involving the affected muscle groups). The median attendance to the resistance training sessions was 23 out of 24 sessions. Of the 32 participants allocated to RTG, 29 (i.e., > 90%) attended ≥ 75% of the exercise sessions. Of them, one reported general discomfort and did not perform the muscular strength assessment at week 12. The adherence to the intensity, repetitions, and volume was exactly as prescribed, except for the above-referenced adverse events. The subjective level of effort for each of the four main resistance training exercises throughout the intervention (i.e., after the familiarization; phase 1) is presented in Supplementary Figure S1. The average number of steps per day during the intervention period was 12,925 (standard deviation [SD] 3951) in the RTG and 12,881 (SD 2352) in the CG. The descriptive characteristics of the study participants by group are presented in Table 1.

**Table 1:** Descriptive characteristics of the study participants overall and by intervention group

	Exercise (*n* = 32)	Control (*n* = 28)
	Mean (SD)	Mean (SD)
Age, years	52.6 (8.8)	52.0 (9.4)
Marital status (married/single/divorced/widow, *n* (%)	26 (81.3)/1 (3.1)/4 (12.5)/1 (3.1)	18 (64.3)/5 (17.9)/5 (17.9)/0 (0)
Educational level (no studies/primary/secondary/university, %)	0 (0)/4 (12.5)/9 (28.1)/19 (59.4)	1 (3.6)/2 (7.1)/7 (25.0)/19 (64.3)
Occupational status (working/housewife/not working, %)	20 (62.5)/4 (12.5)/8 (25.0)	21 (65.0)/1 (3.6)/6 (21.3)
BMI (kg/m^2^)	27.4 (4.2)	26.3 (5.3)
SBP (mm/Hg)	114.8 (16.1)	118.9 (16.4)
DBP (mm/Hg)	69.6 (9.7)	73.0 (11.5)
Current smoking, *n* (%)	3 (9.4)	3 (10.7)
Menopause, *n* (%)	20 (62.5)	19 (67.9)
Time since core treatments ended, years*	3.5 (1–6.75)	4.5 (2–7)
Tumor type, HR + HER2-/HR + HER2 + /HR-HER2 + /HR-HER2- (%)	59.4/18.8/6.3/15.6	71.4/17.9/0.0/10.7
Surgical procedure, *n* (%): tumorectomy/mastectomy	22 (69)/10 (31)	19 (68)/9 (32)
Lymph node resection, *n* (%)	15 (46.9)	10 (35.7)
Endocrine therapy, *n* (%)	27 (84.4)	25 (89.3)
Diagnosed lymphedema, *n* (%)	1 (3%)	5 (18%)
Peak isometric muscular strength		
IMTP (N)	575.3 (255.3)	670.1 (350.9)
Bilateral seated bench press (N)	197.4 (56.2)	185.6 (55.5)
Bilateral seated row (N)	211.6 (56.7)	215.8 (64.2)
Right leg unilateral knee extension in closed kinetic chain at 90° (N)	587.7 (359.9)	576.3 (258.7)
Left leg unilateral knee extension in closed kinetic chain at 90° (N)	670.8 (445.2)	668.9 (293.1)
Right arm unilateral seated bench press (N)	107.6 (22.7)	105.9 (28.0)
Left arm unilateral seated bench press (N)	101.4 (25.1)	98.5 (29.0)
Right arm unilateral seated row (N)	138.2 (31.6)	139.5 (34.9)
Left arm unilateral seated row (N)	134.8 (38.4)	130.6 (38.2)
Handgrip strength affected arm (kg)	25.2 (6.4)	25.4 (5.8)
Handgrip strength nonaffected arm (kg)	26.6 (5.7)	26.5 (5.4)
Estimated VO_2max_ (mL/kg/min)	20.5 (4.7)	21.9 (4.8)
Shoulder flexion (affected arm) (°)	162.0 (19.6)	165.6 (17.7)
Shoulder flexion (nonaffected arm) (°)	172.8 (11.0)	174.3 (9.4)
Cancer-related fatigue (FACT-F total score, 0–52)	40.3 (7.2)	36.4 (10.2)
Depressive symptoms (CES-D total score, 0–60)	15.2 (10.6)	15.5 (11.0)
HRQoL, FACT-B (physical well-being, 0–28)	23.3 (3.8)	22.1 (4.5)
HRQoL, FACT-B (social well-being, 0–28)	22.1 (4.5)	19.7 (5.2)
HRQoL, FACT-B (emotional well-being, 0–28)	17.3 (4.0)	16.5 (4.8)
HRQoL, FACT-B (functional well-being, 0–28)	19.9 (3.6)	17.8 (3.9)
HRQoL, FACT-B (breast cancer subscale, 0–40)	24.3 (4.7)	23.8 (5.2)
HRQoL, FACT-B (total score, 0–148)	107.0 (14.9)	99.9 (17.9)
Satisfaction with Life Scale (total score, 0–25)	18.7 (3.2)	17.8 (4.0)

*SD* standard deviation, *BMI* body mass index, *WHR* waist-to-height ratio, *SBP* systolic blood pressure, *DBP* diastolic blood pressure, *HR* hormone receptor, *HER2*, human epidermal growth factor receptor 2, *IMTP* isometric mid-thigh pull, *N* newtons, *FACT-F* Functional Assessment of Cancer Therapy-Fatigue, *CES-D* Center for Epidemiologic Studies-Depression Scale, *HRQoL* Health-related quality of life, *FACT-B* Functional Assessment of Cancer Therapy-Breast

^*^Median and interquartile range

**Fig. 3:**
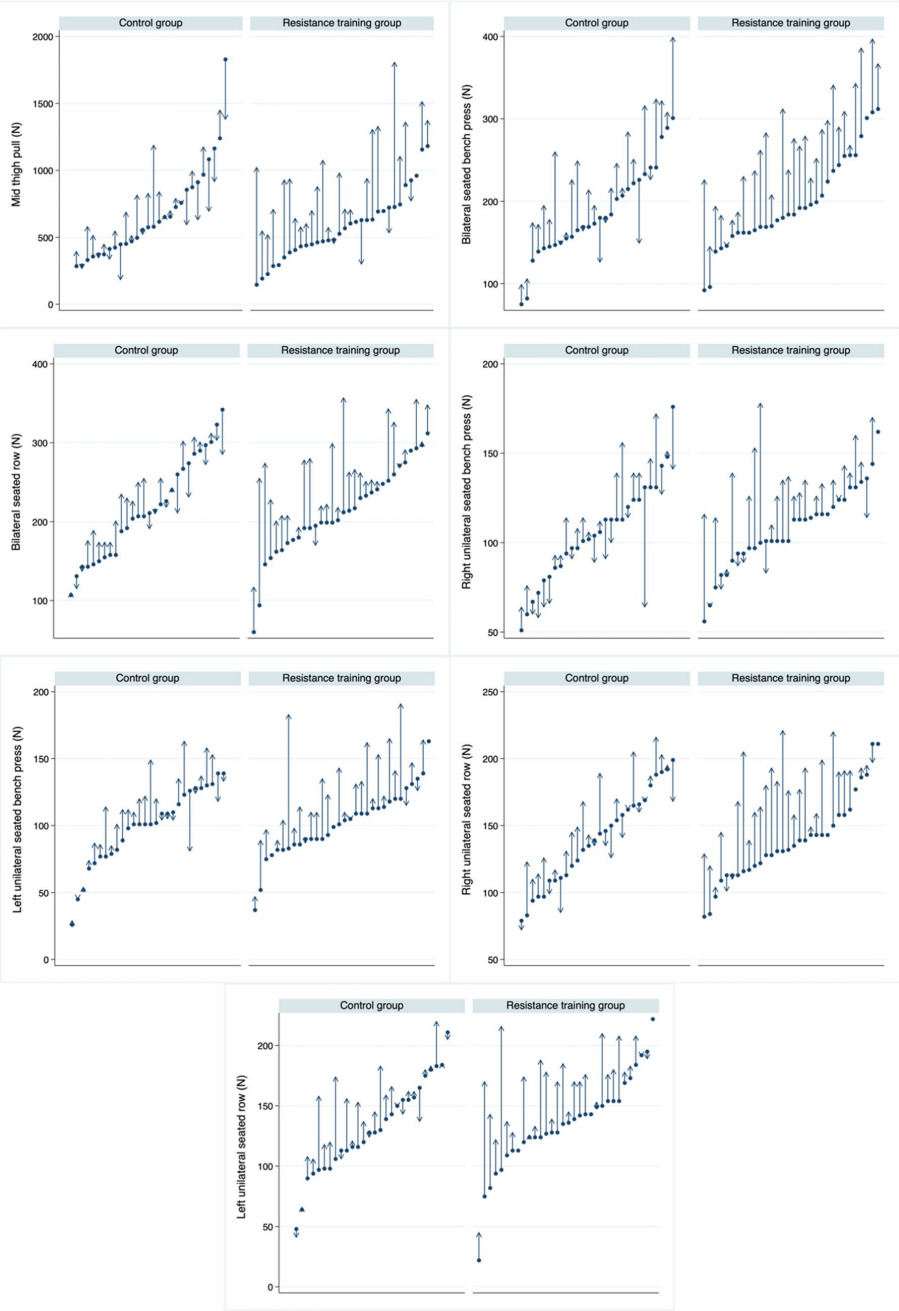
Graphical representation of the effects of the resistance training intervention on the peak isometric muscular strength for the different tests at the individual level in breast cancer survivors. The dots represent the baseline levels, and the arrows represent the changes at 12-week follow-up

The between-group difference in the change from baseline to week 12 in physical fitness and patient-reported outcomes is presented in Tables 2 and 3, respectively. The effects of the resistance training intervention on the peak isometric strength for the different tests at the individual level are presented in Fig. 3.

**Table 2:** Intention-to-treat analyses assessing the effects of the exercise intervention on muscular strength, estimated VO_2max_, and shoulder flexion range of motion in female breast cancer survivors

Change from baseline at week 12	Intervention		Mean difference in the change from baseline to week 12 (95% CI)	Effect size (Cohen’s *d*)	*P*
	Exercise (*n* = 32)	Control (*n* = 28)			
	Mean change (SE)	Mean change (SE)			
Full-body muscular strength, *z*-score	0.335 (0.122)	− 0.383 (0.130)	0.718 (0.361 to 1.074)	1.04	< 0.001
Upper-body muscular strength, *z*-score	0.339 (0.148)	− 0.388 (0.158)	0.727 (0.294 to 1.160)	0.87	0.001
Lower-body muscular strength, *z*-score	0.331 (0.131)	− 0.378 0.140)	0.709 (0.324 to 1.094)	0.96	0.001
IMTP (N)	270.2 (47.2)	49.8 (50.5)	220.4 (81.1 to 359.7)	0.82	0.002
Bilateral seated bench press (N)	60.5 (7.2)	32.3 (7.7)	28.1 (6.9 to 49.4)	0.69	0.010
Bilateral seated row (N)	44.8 (6.3)	9.4 (6.7)	35.5 (17.0 to 53.9)	1.00	< 0.001
Handgrip strength affected arm (kg)	0.7 (0.5)	0.5 (0.5)	0.2 (− 1.3 to 1.6)	0.07	0.811
Handgrip strength nonaffected arm (kg)	0.6 (0.5)	0.6 (0.5)	0.04 (-1.4 to 1.4)	0.00	0.956
Estimated VO_2max_ (mL/kg/min)	0.25 (0.40)	0.83 (0.44)	− 0.57 (− 1.77 to 0.62)	0.25	0.340
Shoulder flexion (affected arm) (°)	− 1.0 (2.2)	0.9 (2.3)	− 2.0 (− 8.3 to 4.4)	0.15	0.540
Shoulder flexion (nonaffected arm) (°)	− 2.2 (2.1)	− 4.2 (2.2)	2.0 (− 4.0 to 8.1)	0.17	0.503

*SE* standard error, *CI* confidence interval, *IMTP* isometric mid-thigh pull, *N* newtons

**Table 3:** Intention-to-treat analyses assessing the effects of the exercise intervention on cancer-related fatigue, depressive symptoms, health-related quality of life, and life satisfaction in female breast cancer survivors

Change from baseline at week 12	Intervention		Mean difference in the change from baseline to week 12 (95%CI)	Effect size (Cohen’s *d*)	*P*
	Exercise (*n* = 32)	Control (*n* = 28)			
	Mean change (SE)	Mean change (SE)			
Cancer-related fatigue, FACT-F, 0–52	1.8 (1.0)	1.4 (1.1)	0.4 (− 2.7 to 3.5)	0.07	0.802
CES-D total score, 0–60	− 2.4 (1.4)	0.0 (1.5)	− 2.4 (− 6.4 to 1.6)	0.30	0.235
FACT-B					
PWB subscale, 0–28	1.5 (0.6)	0.8 (0.6)	0.7 (− 0.9 to 2.4)	0.21	0.374
SWB subscale, 0–28	− 1.9 (0.6)	− 0.5 (0.7)	− 1.4 (− 3.2 to 0.5)	0.40	0.143
EWB subscale, 0–24	0.6 (0.4)	0.1 (0.4)	0.5 (− 0.7 to 1.7)	0.23	0.423
FWB subscale, 0–28	− 0.3 (0.5)	0.0 (0.5)	− 0.3 (− 1.8 to 1.2)	0.11	0.647
BCS subscale, 0–40	0.5 (0.7)	2.0 (0.7)	− 1.5 (− 3.4 to 0.4)	0.39	0.116
FACT-B total score, 0–148	0.0 (1.7)	2.9 (1.8)	− 2.9 (− 7.0 to 2.1)	0.30	0.245
SWLS, 0–25	0.0 (0.4)	− 0.6 (0.5)	0.6 (− 0.7 to 1.9)	0.25	0.344

*SE* standard error, *CI* confidence interval, *FACT-F* Functional Assessment of Cancer Therapy-Fatigue, *CES-D* Center for Epidemiologic Studies-Depression Scale, *FACT-B* Functional Assessment of Cancer Therapy-Breast, *PWB* physical well-being, *SWB* social well-being, *EWB* emotional well-being, *FWB* functional well-being, *BS* breast cancer, *SWLS* Satisfaction with Life Scale

### Primary outcome

The mean changes (SE) in the standardized full-body muscular strength index from baseline to week 12 were 0.335 (0.122) in the RTG and -0.383 (0.130) in the CG (between-group difference 0.718, 95% CI 0.361 to 1.074, *P* < 0.001, *d* = 1.04, Table 2; power (1 – β) = 0.977). These results were consistent in sensitivity analyses (Table S3). Post hoc, we assessed the potential treatment interaction with the time since the core treatments ended, but it was not significant (all *P* > 0.05).

### Secondary outcomes

There were statistically significant group differences in the changes from baseline in all muscular strength outcomes in favor of the RTG, except for the handgrip strength measures (Table 2). There were no group differences in the changes from baseline in any of the other fitness components (Table 2) or the patient-reported outcomes (Table 3).

The sensitivity analyses (Tables S3 and S4) corroborated the results although with relatively larger effect sizes for muscular strength (Table S3).

## Discussion

The main findings of this study indicate that, in female breast cancer survivors who had completed their core treatments within the past 10 years, adding two weekly sessions of supervised resistance training to a prescription of home-based physical activity based on step count for 12 weeks, produced a large increase in upper-, lower-, and full-body muscular strength, although other relevant outcomes such as cancer-related fatigue, depressive symptoms, HRQoL, or life satisfaction did not improve.

We observed that the 12-week supervised resistance training program produced a large increase in the standardized scores for full-body muscular strength (effect size *d* > 1), as well as in the muscular strength of the upper and lower body. This increase in muscular strength was not influenced by the time since the core treatments ended. The muscular strength in the bilateral seated bench press and bilateral seated row increased by 35 and 41 N, respectively, which represents a slightly lower increase in the upper limb compared with the meta-analysis by Strasser et al. [25] who found a weighted mean increase in upper-body strength of muscular strength of 6.9 kg (68 N) following resistance training protocols. By contrast, we found that lower-body muscular strength increased (254 N in the IMTP) substantially more than the weighted average obtained by Strasser et al. (14.6 kg or 143 N [25]). Some of these differences could be partially influenced by the measurement method or the exercise performed during assessments (e.g., seated bench press in comparison to traditional bench press). It is also possible that the larger increase in lower-body strength could be due to the type of resistance training exercises performed during the intervention (i.e., multijoint with free weights for lower-body compared with single joint in guided machines for upper body). However, the resistance exercise intervention followed the exercise principles and had high adherence and good quality of the supervision which could be regarded as relevant aspects that might have had a relevant impact on muscular strength [31]. For instance, we selected four main strength exercises representing major movement patterns that involve muscle groups involved in everyday tasks, and these exercises were also performed during the fitness assessments, therefore maximizing the specificity principle [31] and implying high transference of the intervention into the outcome assessments. Since muscular strength is an important predictor of mortality in breast cancer survivors [27], these results are of clinical relevance and this intervention should be replicated using longer follow-up to determine the extent to which these gains are related to concomitant increases in muscle and bone mass.

The training intervention failed to improve cardiorespiratory fitness and shoulder mobility. Cardiorespiratory fitness has shown to improve following aerobic or combined training interventions in breast cancer survivors. However, in this trial, both groups were recommended to maintain high levels of physical activity to ensure that the international guidelines were met by all. Interestingly, both groups surpassed the average 10,000 steps/day, indicating that both were physically active. However, step count does not relate to exercise intensity and this might explain the lack of improvements in either group regarding cardiorespiratory fitness, where a combined intervention with aerobic training, perhaps of high intensity [65], could have yielded significant fitness improvements. Shoulder mobility (i.e., flexion) was also not improved in the RTG compared to the CG. Unfortunately, we did not assess other movements that have been shown to be impaired following breast cancer treatments, such as abduction, flexion/abduction, and external rotation [12]. Although only 0–9% of patients continue to have reduced range of motion 24 months postsurgery [12], we observed that shoulder flexion was about 10 degrees lower in the affected compared to the unaffected arm at baseline in participants with up to 10 years following treatment. Further research is needed to unravel the extent to which resistance training can enhance shoulder mobility at different stages following treatments [66].

The intervention did not produce a significant improvement in patient-reported outcomes. Regarding cancer-related fatigue, the effects of resistance training in breast cancer survivors are not currently clear. While exercise is globally understood as an efficient therapy against cancer-related fatigue following cancer treatments [67], some relatively large resistance training trials have not observed improvements [68, 69], in line with the results presented here. We might speculate that within three to five years following the core cancer treatments, the baseline levels of cancer-related fatigue might have been reduced, and thus, there would be limited room for improvement. In fact, the average baseline FACT-F total score was well above the proposed value of 34 for diagnosing fatigue [70] in both intervention groups, with only 16 participants out of 60 (10 in the CG and 6 in the RTG) presenting a score of 34 or lower. This rationale could also apply to the other patient-reported outcomes such as depressive symptoms, quality of life, or life satisfaction. For instance, the average CES-D score (~ 15 units in either group) indicated that the participants had no to mild depressive symptomatology [60]. Similarly, the baseline HRQoL of the participants in this study was higher than that observed in other trials [71, 72]. It is likely that a longer intervention including other exercise types such as aerobic/high-intensity training, as well as other behavioral interventions could have yielded further improvements. However, it is also likely that the time frame and the duration of this study might have limited the likelihood of improving certain outcomes, especially considering the CG was physically active.

This study has limitations that must be underlined. First, this is a relatively small study. The participants were compliant, not only with the face-to-face intervention but also with the home-based part. Although this is the strength of the study, the external validity of our results could be threatened by the possibility that the most motivated individuals tended to volunteer participation, which would limit generalizability. Also, we included women who had undergone breast cancer surgery and finished the core treatments up to 10 years before enrolment, which might result in a rather heterogeneous and relatively physically active sample of breast cancer survivors. In addition, we did not collect pre- and postintervention physical activity levels, which might have influenced the results. We cannot ascertain that the precise relative loads (%1RM) the participants trained with throughout the intervention were exactly as they were prescribed, because we used the CE for prescribing the training intensity. Nevertheless, the participants reported an increasing perceived effort as can be observed in Figure S1, suggesting an increasing intensity progression. Further research is needed to determine the equivalence between the perceived effort using the OMNI-RES and the %1RM in breast cancer survivors. However, the intervention was clearly effective as it produced large increases in muscular strength. The major strength of this study is that it accounted for the deficiencies of previous exercise-based clinical trials identified by Neil-Sztramko et al. [31]. We accounted for all key principles of exercise training, reported all components of the exercise prescription in the methods, and reported the adherence to the prescribed intervention in the results. In addition, we provided a comprehensive description of the intervention that is readily available in video format for clinicians or exercise professionals to be used in clinical practice. Thus, the results of this relatively small trial will likely contribute to the development of exercise-oncology prescription [33] for breast cancer survivors.

## Conclusion

In conclusion, the findings of this clinical trial indicate that, in female breast cancer survivors who had completed their core treatments within the past 10 years, a 12-week supervised resistance exercise program combined with home-based physical activity produced a large increase in muscular strength of the upper, lower, and full body compared to home-based physical activity alone. However, we found no group differences in cardiorespiratory fitness, shoulder flexion mobility, cancer-related fatigue, depressive symptoms, HRQoL, and life satisfaction. The patient-reported outcomes may require a longer intervention or follow-up period or the combination of resistance training with diet, aerobic exercise, or other (e.g., psychological) interventions to change. Further research is required to unravel the dose of exercise that provides the greatest benefits for breast cancer survivors.

## Supplementary Information

Below is the link to the electronic supplementary material.

Supplementary file1 (PDF 348 KB)

ESM 1 (PNG 137 kb)

High resolution (TIFF 10084 kb)

Supplementary file2 (ZIP 381 MB)

## Data Availability

The data will be available from the corresponding author upon reasonable request for research purposes.
