# Supplementary material for: Effects of a 12-week supervised resistance training program, combined with home-based physical activity, on physical fitness and quality of life in female breast cancer survivors: the EFICAN randomized controlled trial
Source: J Cancer Surviv. 2022 Mar 22;17(5):1371–85. doi: 10.1007/s11764-022-01192-1 (PMC10442259; doi:10.1007/s11764-022-01192-1)
Supplement: Supplementary file 1 — Supplementary file1 (PDF 348 KB) [file 11764_2022_1192_MOESM1_ESM.pdf]

Table S1. Prescription of the resistance training intervention in terms of frequency, volume, intensity and progressions.

Week	1	2	3	4	5	6	7	8	9	10	11	12
Session	1 2	3 4	5 6	7 8	9 10	11 12	13 14	15 16	17 18	19 20	21 22	23 24
Phases	Phase 1: Familiarization		Phase 2: Circuit Training									
Sets	2	2	2	2	2	3	3	3	3	3	3	2
Repetitions (CE[1] Intensity)	12(30)	12(30)	12(24)	12(24)	12(24)	10(20)	10(20)	10(20)	8(16)	8(16)	8(16)	6(12)
Estimated Intensity (%RM[2])	~40%	~40%	~50%	~50%	~50%	~55%	~55%	~55%	~60%	~60%	~60%	~70%
Total reps per exercise	24	24	24	24	24	30	30	30	24	24	24	12

[1]CE= Number of repetitions to be performed out of the total number of repetitions the participant could perform with the a given load (in brackets).
[2]RM= Repetition Maximum: the weight that can be lifted only once.

Table S2. Exercises performed along the resistance training program*.

		**Exercises**	**Adaptations** (if needed)
**Preparatory part**<br><br>Sets and repetition: 2x8<br><br>Exercise Intensity: -OMNI-RES[2] 3/10 -CE[3] +12	Aerobic activity	5 min of low intensity aerobic activity (50%-65% HRR[1]) on Treadmill	
	Thoracic mobility exercises	Thoracic rotation in sitting position	
		Thoracic extension with fitball in sitting	Perform unilaterally with the unaffected arm
		Column extension from prone in fitball	
		Thoracic rotation in standing	
	Core stability exercises	Pelvic tilt in sitting position	
		Abdominal bracing in sitting position with fitball	Perform unilaterally with the unaffected arm
		Abdominal bracing with band in the transversal plane	If bracing can´t be performed, the position is maintained, and the trainer creates tension in the band
		Bird-dog	Start from wall
	Scapulohumeral joint stability exercises	Humeral rotation without abduction in sitting position	
		Scapulohumeral abduction of 60 ° in the scapular plane	Abduction is replaced for flexion
		Traction (isometric scapulohumeral retropulsion) with 0 ° abduction	
		Internal rotation resisted with band and external rotation resisted with band	Perform in isometry
	Dynamic stability exercises	Bipodal to monopodal in the frontal plane	Perform with a safety point in one hand
		Lateral shift	Perform with a safety point in one hand
		Slow skipping	Start from wall
		Go up and down to a step with stop monopodal	Perform with a safety point in one hand
**Resistance training**	Strength	Bilateral Dead Lift	Progress from the last part of the exercise (rack pull) to the initial, increasing the ROM progressively
		Bilateral Seated Row	If it can´t be done by dorsal flap reconstruction, perform unilaterally with the unaffected arm
		Bilateral Squat	Increasing the ROM progressively
		Bilateral Seated Bench Press	If it can´t be done by dorsal flap reconstruction, perform unilaterally with the unaffected arm
**Cool-Down**	Dynamic/static stretching of major muscle groups	Pectoralis major, Dorsal Width, Quadriceps, Hamstrings	

Sets and repetition: 1x12			

[1]HRR: heart rate reserve; [2]OMNI-RES: Perceived Exertion Scale for Resistance Exercise (0-10); [3]CE Number of repetitions that the participant could performed with the actual weight.

* Note that not all these exercises will be performed in the same session. Rather, this represent the spectrum of exercises that are to be used along the training program.

Extended description of the standardized full-body muscular strength index.

First, a z-score (z-score = [value−mean] / standard deviation) was computed for the change from baseline to week 12 in each of these tests:

- Seated bench press
- Seated row
- Isometric mid-thigh pull (IMTP)
- Knee extension in closed kinetic chain at 90°

The seated bench press z-score was computed as follows: The change from baseline to week 12 in the muscular strength of the right and left sides was added. A z-score for this sum was computed.

The seated row z-score was computed as follows: The change from baseline to week 12 in the muscular strength of the right and left sides was added. A z-score for this sum was computed.

The IMTP z-score was computed as follows: A z-score of the change from baseline to week 12 in the isometric mid-thigh pull (IMTP) test was computed.

The knee extension in closed kinetic chain at 90° z-score was computed as follows: The change from baseline to week 12 in the muscular strength of the right and left sides was added. A z-score for the sum was computed.

Finally, the standardized full-body muscular strength index was computed as the average of the 4 above-mentioned z-scores.

Table S3. Per-protocol (sensitivity) analyses assessing the effects of the exercise intervention on muscular strength, estimated VO_{2max} and shoulder flexion range of motion in female breast cancer survivors, considering participants who attended ≥75% of the exercise sessions.

Change from baseline at Week 12	Intervention		Mean difference in the change from baseline to week 12 (95%CI)	Effect size (Cohen's *d)*	P
	Exercise (n=28)	Control (n=28)			
	Mean change (SE)	Mean change (SE)			
Full-body muscular strength, z-score	0.427 (0.124)†	-0.422 (0.121)	0.849 (0.501 to 1.197)	-1.32	<0.001
Upper-body muscular strength, z-score	0.398 (0.154)	-0.423(0.154)	0.821 (0.384 to 1.257)	-1.01	<0.001
Lower body muscular strength, z-score	0.417 (0.135)†	-0.421 (0.133)	0.839 (0.458 to 1.219)	-1.19	<0.001
IMTP, N	303.7 (51.5)†	49.6 (50.5)	254.1 (108.4 to 399.9)	-0.94	0.001
Bilateral seated bench press, N	67.1 (7.5)	32.4 (7.5)	34.7 (13.6 to 55.9)	-0.87	0.002
Bilateral seated row, N	50.2 (6.7)	9.8 (6.7)	40.5 (21.5 to 59.4)	-1.14	<0.001
Handgrip strength affected arm, kg	0.7 (0.5)	0.5 (0.5)†	0.15 (-1.5 to 1.7)	-0.08	0.843
Handgrip strength non-affected arm, kg	0.5 (0.5)	0.6 (0.5)†	-0.1 (-1.5 to 1.4)	0.04	0.914
Estimated VO_{2max}, mL/kg/min	0.51 (0.43)	0.83 (0.45)‡	-0.33 (-1.58 to 0.92)	0.14	0.601
Shoulder flexion (affected arm), º	-0.9 (2.3)	0.9 (2.4)	-1.8 (-8.4 to 4.9)	0.14	0.596
Shoulder flexion (nonaffected arm), º	-2.4 (2.2)	-4.3 (2.3)	1.8 (-4.6 to 8.1)	-0.16	0.586

SE, standard error; CI, confidence interval; IMTP, isometric mid-thigh pull; N, newtons.
† (n=27); ‡ (n=26)

Table S4. Per-protocol (sensitivity) analyses assessing the effects of the exercise intervention on cancer-related fatigue, depressive symptoms, health-related quality of life and life satisfaction in female breast cancer survivors.

Change from baseline at Week 12	Intervention		Mean difference in the change from baseline to week 12 (95%CI)	Effect size (Cohen's *d)*	P
	Exercise (n=29)	Control (n=27)			
	Mean change (SE)	Mean change (SE)			
Cancer-related fatigue, FACT-F, 0-52	1.9 (1.1)	1.5 (1.2)	0.4 (-2.9 to 3.7)	-0.07	0.803
CES-D total score, 0-60	-2.3 (1.4)	0.1 (1.5)	-2.4 (-6.6 to 1.8)	0.31	0.253
FACT-B					
HRQoL, PWB subscale, 0-28	1.6 (0.6)	0.9 (0.6)	0.7 (-1.0 to 2.5)	-0.22	0.405
HRQoL, SWB subscale, 0-28	-1.9 (0.7)	-0.5 (0.7)	-1.4 (-3.3 to 0.6)	0.38	0.162
HRQoL, EWB subscale, 0-24	0.7 (0.4)	0.1 (0.5)	0.6 (-0.7 to 1.8)	-0.25	0.362
HRQoL, FWB subscale, 0-28	-0.5 (0.5)	0.0 (0.6)	-0.6 (-2.1 to 1.0)	0.17	0.473
HRQoL, BC subscale, 0-40	0.6 (0.7)	2.0 (0.7)	-1.5 (-3.5 to 0.5)	0.38	0.145
FACT-B total score, 0-148	0.0 (1.8)	3.0 (1.9)	-2.9 (-8.3 to 2.4)	0.31	0.275
SWLS, 0-25	0.0 (0.5)	-0.7 (0.5)	0.7 (-0.7 to 2.0)	-0.26	0.330

SE, standard error; CI, confidence interval; FACT-F, Functional Assessment of Cancer Therapy-Fatigue; CES-D, Center for Epidemiologic Studies-Depression Scale; FACT-B, Functional Assessment of Cancer Therapy-Breast; SWLS, Satisfaction With Life Scale.

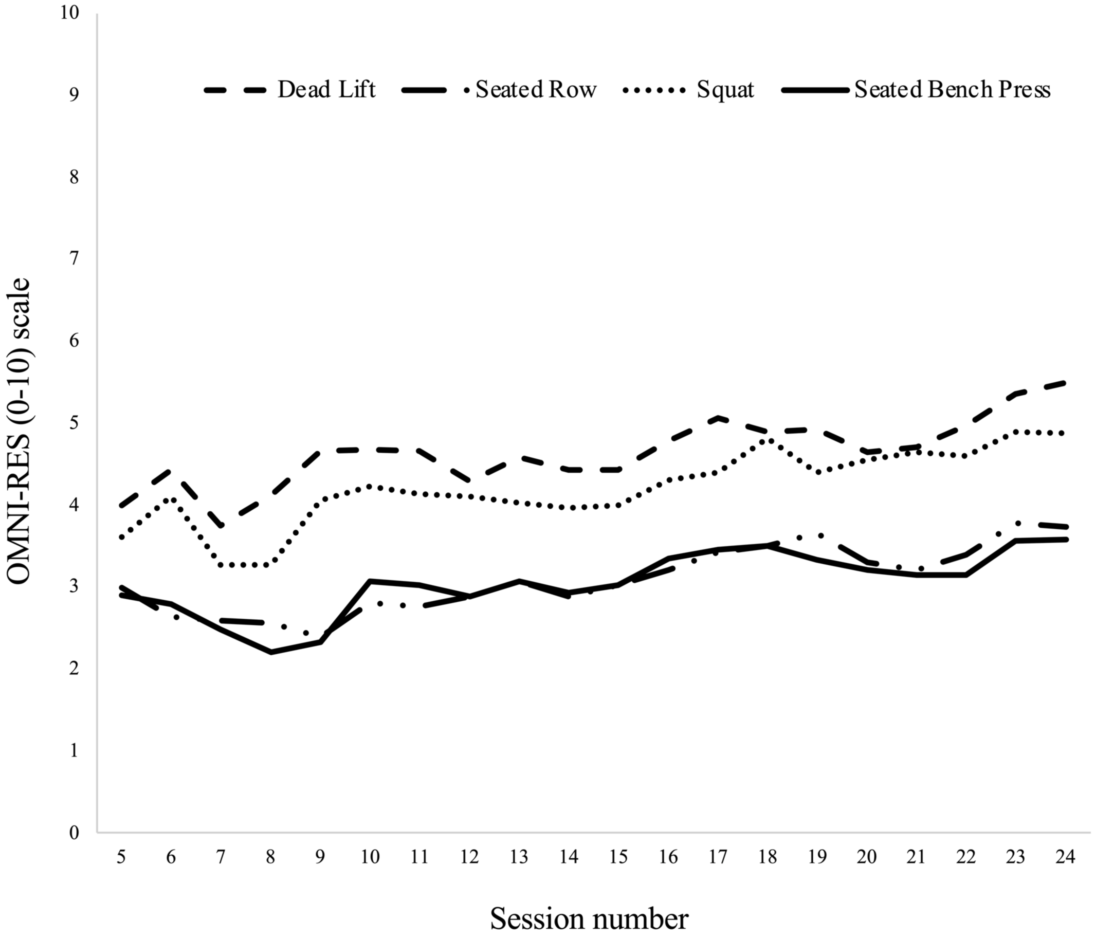

Figure S1. Perceived effort for each of the 4 main resistance exercises throughout the intervention, assessed through the OMNI Perceived Exertion Scale for Resistance Exercise (OMNI-RES).
