# Supplementary figures and images for: Effects of a 12-week supervised resistance training program, combined with home-based physical activity, on physical fitness and quality of life in female breast cancer survivors: the EFICAN randomized controlled trial
Source: J Cancer Surviv. 2022 Mar 22;17(5):1371–85. doi: 10.1007/s11764-022-01192-1 (PMC10442259; doi:10.1007/s11764-022-01192-1)

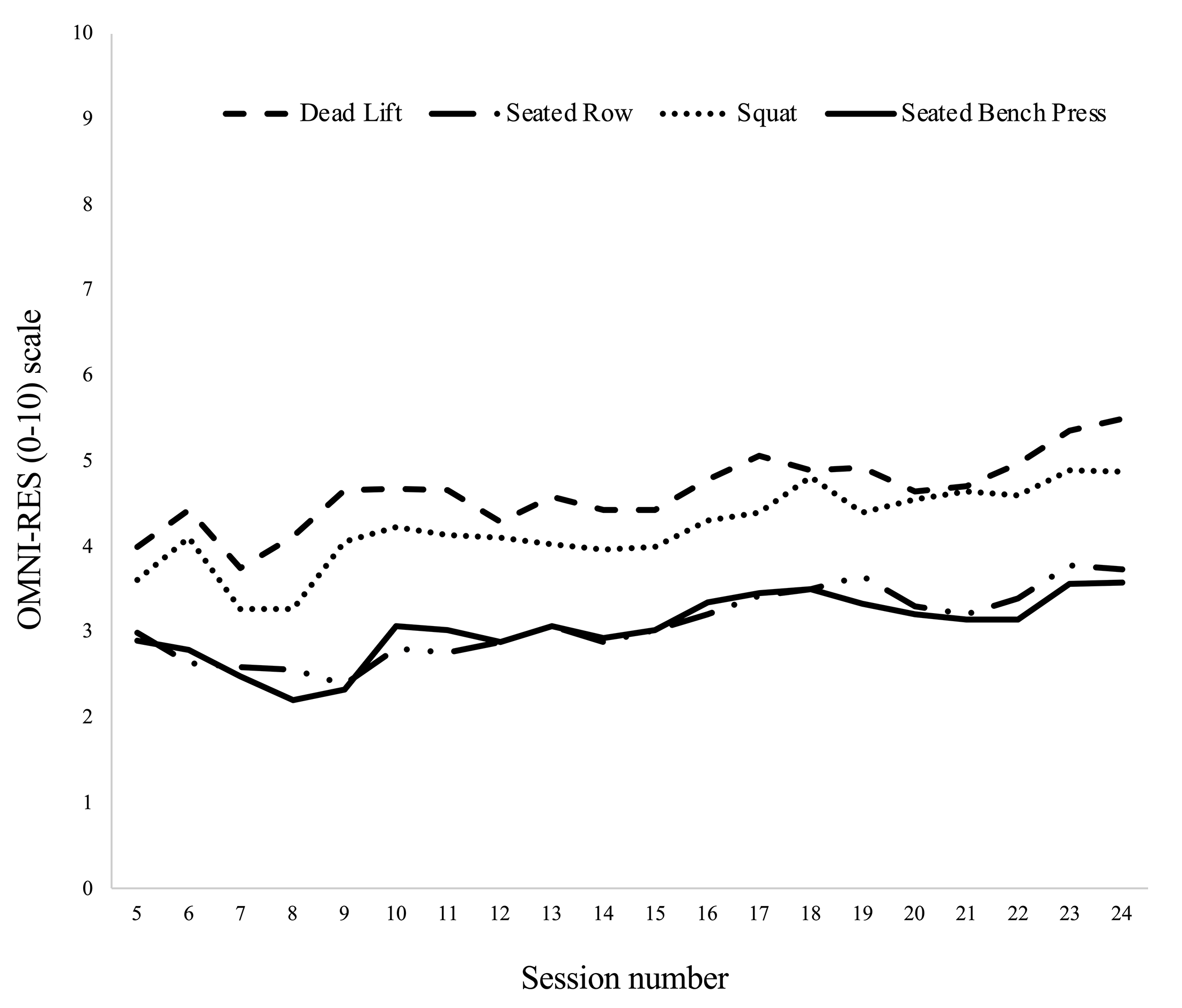

Supplement: Supplementary file 2 — (PNG 137 kb) [file 11764_2022_1192_Fig4_ESM.png]

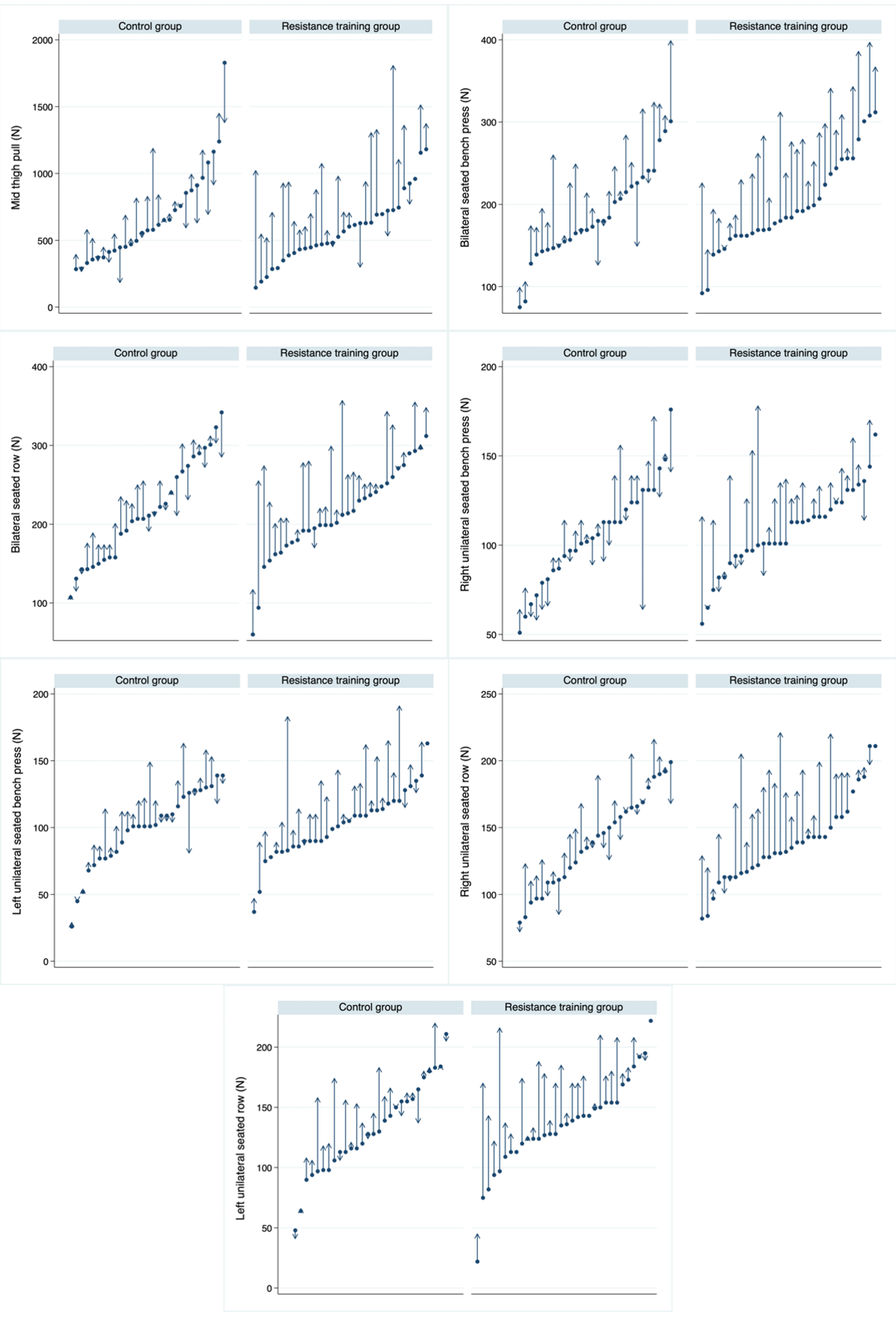

Supplement: Supplementary file 3 — High resolution (TIFF 10084 kb) [file 11764_2022_1192_MOESM3_ESM.tiff]
